# Self‐Healing of Electrical Damage in Polymers

**DOI:** 10.1002/advs.202002131

**Published:** 2020-09-30

**Authors:** Yang Yang, Zhi‐Min Dang, Qi Li, Jinliang He

**Affiliations:** ^1^ State Key Laboratory of Power System Department of Electrical Engineering Tsinghua University Beijing 100084 China; ^2^ Present address: Simpson Querrey Institute Northwestern University Evanston IL 60208 USA

**Keywords:** dielectric polymers, electrical breakdown, electrical insulation, electrical trees, self‐healing

## Abstract

Polymers are widely used as dielectric components and electrical insulations in modern electronic devices and power systems in the industrial sector, transportation, and large appliances, among others, where electrical damage of the materials is one of the major factors threatening the reliability and service lifetime. Self‐healing dielectric polymers, an emerging category of materials capable of recovering dielectric and insulating properties after electrical damage, are of promise to address this issue. This paper aims at summarizing the recent progress in the design and synthesis of self‐healing dielectric polymers. The current understanding to the process of electrical degradation and damage in dielectric polymers is first introduced and the critical requirements in the self‐healing of electrical damage are proposed. Then the feasibility of using self‐healing strategies designed for repairing mechanical damage in the healing of electrical damage is evaluated, based on which the challenges and bottleneck issues are pointed out. The emerging self‐healing methods specifically designed for healing electrical damage are highlighted and some useful mechanisms for developing novel self‐healing dielectric polymers are proposed. It is concluded by providing a brief outlook and some potential directions in the future development toward practical applications in electronics and the electric power industry.

## Graphical abstract

Recent advancements in self‐healing dielectric polymers are reviewed. The complex mechanisms and characteristics of electrical damage in polymers bring various challenges of applying conventional self‐healing approaches to repair electrical damages in polymers. Besides several emerging approaches to restore insulating properties upon electrical damages, future directions in developing self‐healing insulation devices and systems in practical applications are discussed.

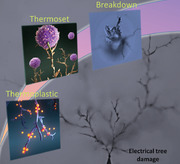

## 1 Introduction

**Figure 1:**
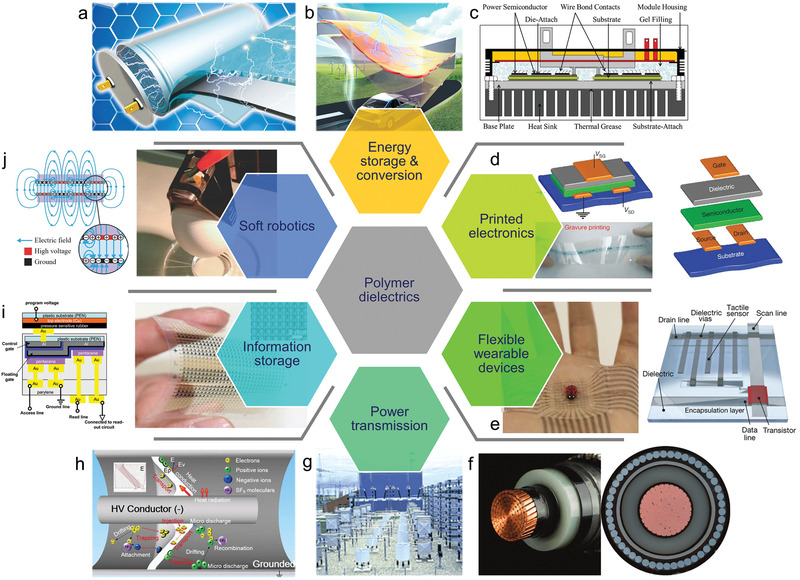
Main application fields of dielectric polymers.a–c) Energy storage and conversion including film capacitors and power electronics acting as essential elements in electric and hybrid vehicles. a) Reproduced with permission.^[^ ^5^ ^]^ Copyright 2015, Royal Society of Chemistry. b) Reproduced with permission.^[^ ^6^ ^]^ Copyright 2019, Wiley‐VCH. c) Reproduced with permission.^[^ ^7^ ^]^ Copyright 2016, Institute of Electrical and Electronic Engineers. d) Printed electronics such as organic thin‐film transistors. Reproduced with permission.^[^ ^11^ ^]^ Copyright 2009, Nature Publishing Group. e) Flexible wearable devices such as skin electronics. Reproduced with permission.^[^ ^13^ ^]^ Copyright 2018, Nature Publishing Group. f–h) Power transmission installations including gas‐insulated transmission lines/substations and extruded power cables. Reproduced with permission.^[^ ^23^, ^28^, ^29^ ^]^ Copyright f) 2007, g) 2013, h) 2018, Institute of Electrical and Electronic Engineers. i) Information storage devices such as the organic floating‐gate transistor sheet. Reproduced with permission.^[^ ^15^ ^]^ Copyright 2009, American Association for the Advancement of Science. j) Soft robotics such as dielectric elastomer actuators. Reproduced with permission.^[^ ^19^ ^]^ Copyright 2016, Wiley‐VCH.

Polymers such as low‐density polyethylene (LDPE), cross‐linked polyethylene (XLPE) polypropylene (PP), polyimide (PI), epoxy, polydimethylsiloxane (PDMS), and polyvinylidene fluoride (PVDF) are dielectric materials with high breakdown strength, great electrical resistivity, light weight, and low manufacturing cost, which have been extensively used in electric energy storage,^[^ ^1^, ^2^, ^3^, ^4^, ^5^, ^6^ ^]^ power electronics,^[^ ^7^, ^8^ ^]^ printed circuits,^[^ ^9^, ^10^, ^11^ ^]^ wearable/skin inspired electronics,^[^ ^12^, ^13^, ^14^ ^]^ information storage devices,^[^ ^15^, ^16^ ^]^ soft robotics,^[^ ^17^, ^18^, ^19^, ^20^ ^]^ and high voltage electrical insulations (**Figure** **1**).^[^ ^21^, ^22^, ^23^, ^24^ ^]^ Failure of electronic devices and electrical equipment usually occurs in the dielectric and insulation elements withstanding strong electric field and/or supporting conductors of high electric potential.^[^ ^25^, ^26^, ^27^, ^28^, ^29^ ^]^ Despite that the electrical insulating properties of gas or liquid materials are highly restorable after electrical damage and that some semi‐liquid semi‐solid silicone gels exhibit self‐healing behaviors, electrical damage in solid polymers has long been recognized as permanent defects.^[^ ^30^ ^]^ In the meantime, the rapidly rising need of electricity and widespread usage of consumer electronics both demand advanced dielectric polymers with substantially improved reliability and lifespan. Therefore, dielectric and insulating polymers with self‐healing functionalities are becoming an emerging research field that arouses broad interest.

By mimicking biological systems that are capable of autonomously healing wounds for better survival, self‐healing materials usually introduce healing components into the host matrix (extrinsic approaches) or employ reversible chemical bonds (intrinsic approaches) to rebuild the damaged regions and hence gain extended service lifetime. Although various self‐healing materials have been designed to repair mechanical damage (cut, scratch, rupture, puncture, etc.),^[^ ^31^, ^32^, ^33^, ^34^, ^35^, ^36^ ^]^ directly applying those approaches to electrical insulating applications would face various difficulties. This is because the mechanism and damaging process of electrical degradation are fundamentally different from the mechanical damage. A typical electrical damaging event of dielectric polymers is sophisticated, and it involves the electrical treeing process accompanied with electrochemical, photochemical and thermochemical degradation, followed by the catastrophic breakdown.^[^ ^22^, ^25^, ^37^ ^]^ The electrical treeing phenomenon gives rise to the formation of dendric hollow cracks of few micrometers in the tube diameter, which usually fall into the gap between the damaging scales that intrinsic (from molecular‐ to micrometer‐scales) and extrinsic (larger than tens of micrometers) approaches can heal (see details in **Table** **1**, Section 3). In addition, most of the existing self‐healing materials have insufficient electrical resistivity and breakdown strength that inhibit their use in the proposed dielectric and insulation applications including power cables, electronic modules, high‐voltage capacitors, etc. (see details in **Table** **2**, Section 3).

**Table 1:** Characteristics of some self‐healing methods that possess potential applications in dielectric polymers and functional requirements of self‐healing dielectric polymers

Self‐healing Mechanisms and Electrical Damage		Most Effective Healing Scale Range	Healing Cycles	Waiting Time	Chemical Degradation Tolerance	Healing Conditions
Extrinsic Methods	Single‐component Microcapsule	10–100 µm	Single	Long, depends on the storage lifetime of the healing agent.	High, depends on the storage of the healing agent.	24 h, ambient 1 h, 220 °C
	Hollow Fibers & Vascular Network	Micro‐ to Millimeters	<30			2–48 h, ambient 1–24 h, 70–100 °C
Intrinsic Methods	Hydrogen Bond	Molecular scale (in contact) and depends on liquidity, usually <1 µm.	Highly repeatable	Approximately hours, surface deactivation in air.	Low, deactivation by electrical degradation.	Ambient pressure: approximately minutes
	Other Reversible Noncovalent					Ambient: approximately minutes UV, pressure: approximately hours
	Reversible and Irreversible Covalent		<10			Heating: 5 min to 24 h RT, UV: 0.5 h to 10 days
	Ionomers	approximately Millimeters	N/A	≈0	N/A	Ambient, rapid impact
Electrical Damage	Initial Tree	Diameter: nanoscale Length: few microns	Repeated damaging due to inherent defects	Long waiting time is needed.	Little chemical degradation	Bulk dielectrics operate under high voltage (field), high temperature. Generating free radicals, UV electroluminescence.
	Propagating Tree	Diameter: 1–10 µm Length: >0.1 mm			Some chemical degradation	
	Ultrathin Film Breakdown	Nanoscale, depending on film thickness, etc.		Not needed if breakdown events can be detected.	Little chemical degradation	Power app.: high‐field and high temperature. Electronics: low‐field, RT. Generate Maxwell stress, heat, carbonization, etc.
	Bulk Breakdown	Tens of microns or larger			Severe chemical degradation	

**Table 2:** Typical mechanical and electrical properties of existing insulating self‐healing polymers and conventional high‐field dielectric polymers

Insulating Self‐healing Materials & Dielectric polymers	Young's Modulus	*ε*_r_, [1 kHz]	DC Breakdown Strength, (*E*_b_/10)	Electrical Resistivity	*T*_g_	Dielectric Applications
Microcapsule or Vascular Composites	0.8–3.4 GPa	4 ^[^ ^62^ ^]^	30–40 MV m^−1^, ^[^ ^62^ ^]^ (288–594 MV m^−1^)	10^15^–10^16^ Ω cm ^[^ ^62^ ^]^	≈127 °C	Cable termination, bushing, etc.
Hydrogen Bond [Zigzag H‐bond]^[^ ^126^ ^]^	<100 MPa [<2 GPa]	No general value is reported.	(<204 MV m^−1^)^a)^ [N/A, (<910 MV m^−1^)^a)^]	No general value is reported.	Approximately RT [<60 °C]	Electroactuators & flexible electronics (Low electric field, room temperature)
Other Reversible Noncovalent	<680 kPa		(<16.8 MV m^−1^)^a)^		Approximately RT	
Reversible and Irreversible Covalent	<80 MPa		(<182 MV m^−1^)^a)^		Healing temperature	
Ionomers (Surlyn)	<350 MPa	3.8	130 µm: ≈90 MV m^−1^,^b)^ (< 195 MV m^−1^)	10^13^–10^14^ Ω cm^[^ ^127^ ^]^	≈50 °C	Moderate temperature
Epoxy	3–5 GPa	3.2	1 mm: 165 MV m^−1^ (624–805 MV m^−1^)	10^16^ Ω cm	137 °C	Bulk: moderate and high temperature.
XLPE	1 GPa	2.4	150 µm: 270 MV m^−1^ (407 MV m^−1^)	10^16^–10^17^ Ω cm	90 °C	
Linear LDPE	137–520 MPa	2.3	250 µm: 350 MV m^−1^, (157–306 MV m^−1^)	10^16^ Ω cm	<0 °C	Bulk and thin film: moderate temperature.
Isotactic PP	1.4 GPa	2.3	733–800 MV m^−1^ (BOPP), (502 MV m^−1^)	10^16^–10^17^ Ω cm	≈0 °C	
PI (Kapton)	2.5 GPa	2.7–3.5	8–13 µm: ≈600 MV m^−1^, (544–620 MV m^−1^)	2.3 × 10^17^ Ω cm	360–410 °C	Thin film: high temperature.
PEI^c)^ (Ultem)	3.0 GPa	3.15	<20 µm: >600 MV m^−1^, (628 MV m^−1^)	10^17^ Ω cm	217–247 °C	
PPS^d)^ (Torelina)	3.5–4.7 GPa	3	9 µm: 490 MV m^−1^, (695–806 MV m^−1^)	10^16^ Ω cm	118 °C	
PEEK^e)^ (KetaSpire)	3.5–3.7 GPa	3.1	8 µm: 480–590 MV m^−1^, (684–704 MV m^−1^)	2.6 × 10^16^ Ω cm	150 °C	

^a)^ No general dielectric constant is reported, *ε*_r_ = 1 in calculation of *E*_b_;

^b)^ NASA Technical Report Server, Report/Patent Number: GRC‐E‐DAA‐TN53154;

^c)^ Poly(ether imide);

^d)^ Poly(phenylene sulfide);

^e)^ Poly(ether ether ketone).

Self‐healing materials designed for repairing mechanical damage have been previously reviewed from different perspectives such as material classification (conductive and metallic,^[^ ^38^, ^39^ ^]^ graphene‐based,^[^ ^40^ ^]^ microcapsule‐based and vascular networks,^[^ ^41^, ^42^ ^]^ dynamic chemical bonding)^[^ ^43^, ^44^ ^]^ and potential application area (structural materials,^[^ ^45^ ^]^ coatings,^[^ ^46^, ^47^ ^]^ energy harvesting and storage devices,^[^ ^48^, ^49^ ^]^ flexible electronics,^[^ ^50^ ^]^ soft robotics,^[^ ^51^ ^]^ medical materials,^[^ ^45^ ^]^ etc.). However, none of them discussed about self‐healing of electrical damage in polymers. On the other hand, in the field of dielectric polymers, most of the existing studies have focused on improving breakdown strength and thermal stability,^[^ ^4^ ^]^ modulating dielectric constant,^[^ ^52^ ^]^ reducing dielectric loss ^[^ ^1^, ^53^ ^]^ and exploring other functionalities.^[^ ^18^, ^54^, ^55^ ^]^ Research on electrical tree damage has been limited to improving the tree inception voltage and restraining the tree propagation by adding voltage stabilizers,^[^ ^56^, ^57^ ^]^ degradation inhibitors,^[^ ^58^ ^]^ nano‐/micro‐scale fillers,^[^ ^59^, ^60^ ^]^ etc., rather than healing the damage or recovering the insulating property. After a long period of time's exploration and development, recently, self‐healing of electrical tree damage and complete restoration of insulating properties in bulk dielectric polymers were attained.^[^ ^61^, ^62^, ^63^ ^]^ We believe this is the right time to implement a review of the achievement by far in this emerging field, which would serve to inspire future work in self‐healing dielectric polymers.

In this paper, we begin with an overview on the current understanding to the characteristics of electrical damage in polymers (Section 2). Then the possibility of applying conventional self‐healing approaches to the repair of electrical damages in dielectric polymers is discussed, followed by a summary of challenges and bottleneck issues in developing self‐healing dielectric polymers (Section 3). Afterward, some emerging studies on self‐healing of electrical tree and breakdown damages are reviewed in detail in Section 4. In Section 5, some useful mechanisms for novel self‐healing dielectric polymers are proposed on the basis of the accompanying processes in electrical damaging and the limitations in the existing self‐healing dielectric polymers. Finally, a brief summary and outlook is presented regarding the possible improvement of existing self‐healing dielectric polymers and future directions in developing self‐healing insulation devices and systems for various practical applications.

## 2 Electrical Damage in Dielectric Polymers

**Figure 2:**
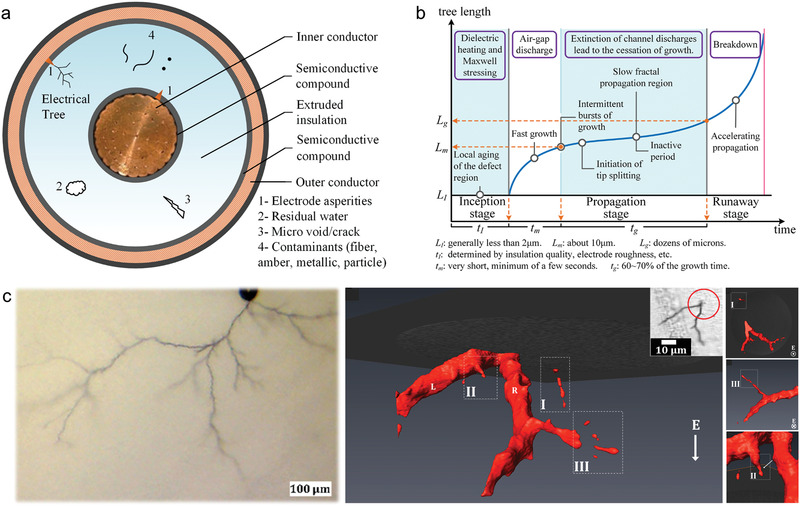
Electrical tree damages in dielectric polymers. a) Typical initial defects in extruded polymer power cable. b) Statistic trend of tree length as a function of propagation time based on the reported researches in ref. [25] Two inactive periods, the inception stage and the slow propagation region, can be utilized to stop the treeing process and heal the damages. c) Optical microscopic image (left) and 3D rendering by computed micro‐X‐ray tomography (right) of the electrical tree in polyethylene. Reproduced with permission.^[^ ^75^ ^]^ Copyright 2017, American Chemical Society.

Research on electrical tree aging and breakdown of polymers dates back to early the 20th century.^[^ ^25^, ^64^ ^]^ To date, mechanisms and physical processes of electrical aging have not yet been fully understood, bringing many challenges to the design of self‐healing dielectrics. Electrical treeing is the primary origin of insulation failure in bulk dielectric polymers,^[^ ^25^ ^]^ which is usually initiated from insulation defects and propagates rapidly in the form of microscale dendric hollow cracks till catastrophic breakdown and collapse of the whole material. Taking the extruded power cable as an example, as shown in **Figure** **2**a, the insulation defects (pre‐existing or formed during service) include physical defects of microscale void/cracks and chemical defects of impurities. The clearing of pre‐existing, inherent defects in polymers is still an open issue. Herein, our discussion mainly focuses on the healing of defects initiated during the service under electrical stress, i.e., electrical damage.

According to the tree propagation rate in different periods of electrical damage, the treeing process can be divided into three stages as illustrated in Figure 2b, i.e., I) Inception stage, II) Propagation stage (including fast growth and slow fractal propagation periods), and III) Runaway stage.

Electrical treeing is initiated by the defect regions, as illustrated in Figure 2a, which are pre‐existing in the dielectrics or generated by chemical degradation and Maxwell stress.^[^ ^65^, ^66^ ^]^ Under high electric fields, space charge injection and recombination (especially near the electrode asperities)^[^ ^67^ ^]^ will accelerate the tree formation process initiated from the defect regions.^[^ ^68^ ^]^ Above the inception field,^[^ ^69^ ^]^ electrical tree propagation is driven by partial discharge events in the tree channels, with the initiation voltage depending on the material type and the shape of electrode asperities.^[^ ^70^, ^71^ ^]^ After the inception stage, the treeing process usually features decelerated propagation^[^ ^65^ ^]^ and shows intermittent, stepwise bursts of growth.^[^ ^27^, ^72^ ^]^ Then it goes into a relatively slow propagation period (or inactive period) accompanied with tip splitting.^[^ ^25^ ^]^ The formation of branch channels weakens the local electric field at the tree tips and may lead to the cessation of tree growth.^[^ ^73^ ^]^ For continued propagation, the tree shows accelerated growth as the forefront branches approach the opposite electrode. Finally, in the runaway stage the accelerated growth turns into catastrophic breakdown, and a micro‐ to millimeter scale through hole is formed.^[^ ^65^, ^74^ ^]^

Taking into account the competition between the electrical damage and healing process, i.e., the formation and propagation process of electrical treeing versus the healing rate of the existing self‐healing methods, two periods in the electrical treeing can be utilized to heal the damage: the inception stage and the slow propagation stage. In the inception stage, nano‐scale damage (e.g., chain rupture) is involved and the healing methods capable of dealing with molecular‐ to nano‐level cracks would be effective. In the propagation stage when dendric hollow tree channels are formed with diameter of few micrometers and length of hundreds of micrometers or longer, the self‐healing approaches employed should be able to mend micrometer‐ to millimeter‐scale damage. Actually, the electrical tree is a multiscale damage containing microscale cracks and nanoscale voids at tree branch ends,^[^ ^75^ ^]^ as shown in Figure 2c, and a better strategy for the self‐healing should cover a broad range of length scales from nanometer to millimeter.

Considering the special working conditions of dielectric materials and the unpredictable occurrence of electrical damage, ideal self‐healing dielectric polymers should satisfy the basic requirements as follows:

1. The self‐healing dielectric polymers should possess dielectric properties comparable to the conventional polymer insulations, including breakdown strength, dielectric loss, electrical resistivity, etc.
2. The self‐healing systems should be sensitive to multiple length scale of defects.
3. The healing process must be quicker than the damaging process, e.g., the propagation rate of electrical trees.

## 3 Challenges in Developing Self‐Healing Dielectric Polymers

### 3.1 Tackling the Electrical Damage with Conventional Self‐Healing Strategies

In this section, existing self‐healing strategies originally designed for repairing mechanical damage are briefly reviewed, and previous tentative research applying those strategies to the healing of electrical damage in bulk dielectric polymers are discussed. According to the healing mechanism, the existing self‐healing strategies can be roughly divided into two categories: 1) extrinsic self‐healing via embedded healing agent carried by microcapsules, hollow fibers, microvascular, etc., and 2) intrinsic self‐healing without additional healing agent, such as non‐covalent interactions, reversible covalent bonds, and irreversible covalent bonds.

#### 3.1.1 Extrinsic Self‐Healing Methods

**Figure 3:**
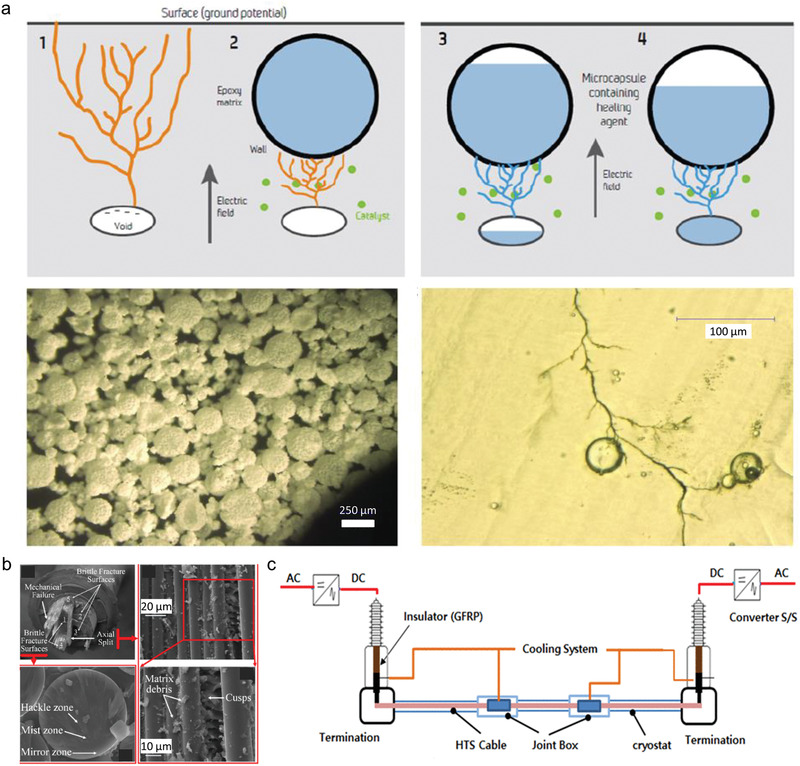
Previous tentative research and possible applications for extrinsic self‐healing dielectric polymer. a) Healing of electrical tree damages using microcapsules in epoxy matrix. Reproduced with permission.^[^ ^77^ ^]^ Copyright 2014, Institute of Electrical and Electronic Engineers. b) Glass fiber reinforced polymer rod in high voltage composite insulator. Reproduced with permission.^[^ ^84^ ^]^ Copyright 2018, Institution of Engineering and Technology. c) Conceptual structure of high‐temperature superconducting dc cable system using glass fiber reinforced polymer termination. Reproduced with permission.^[^ ^85^ ^]^ Copyright 2016, Institution of Electrical and Electronic Engineers.

For extrinsic self‐healing approaches, liquid healing agents embedded in the polymer matrix are released into the damaged regions by capillary effect when the carriers are ruptured by the damage, which then polymerize upon contacting the metal‐containing catalysts doped in the matrix.^[^ ^76^ ^]^ Lesaint et al. tried to utilize conventional Grubbs’ catalyst‐based microcapsule self‐healing method to heal electrical trees in epoxy,^[^ ^77^ ^]^ as shown in **Figure** **3**a. However, the electrical tree channels are usually quite slim (nanometer scale to several microns in diameter) and are of limited branch lengths (below or at millimeter scale) before their growth steps into the uncontrolled stage, and therefore the chance for the tree cracks to engage the catalyst is low, yielding much lower healing efficiency in comparison with the healing of mechanical cracks. In addition, incorporation of metal‐containing catalyst would deteriorate the insulating properties of the dielectric polymers, bring in additional insulation defects ^[^ ^78^ ^]^ and increase the probability of electrical tree inception. Various single‐component extrinsic self‐healing systems without catalysts have been developed utilizing the healing agents that can cure under external stimulations or upon contacting other reactants,^[^ ^79^, ^80^, ^81^ ^]^ providing opportunities for developing catalyst‐free self‐healing dielectric polymers. But whether or not the healing kinetics can surpass that of the electrical damage process needs to be evaluated.

In many electrical insulation applications requiring high mechanical performance such as fiber‐reinforced composite insulators,^[^ ^82^, ^83^, ^84^ ^]^ cable termination and bushing (Figure 3b,c),^[^ ^85^, ^86^ ^]^ and power electronics packaging,^[^ ^7^ ^]^ extrinsic self‐healing mechanisms can be adopted by replacing the embedded solid fibers with hollow fibers containing healing agents.^[^ ^87^ ^]^ The challenge in designing such composites is that the fibers must be “fragile” enough so that they can be ruptured during the electrical damage, and, in the meantime, be “strong” enough to enhance the mechanical strength of the composites. Since the existence of intrinsic material defects, multiple electrical tree damage may occur in the same region, and therefore the ability of repeated healing is required, which is usually absent in the extrinsic approaches. Although microvascular self‐healing systems feature highly repeatable healing ability,^[^ ^88^ ^]^ the design and fabrication of complex 3D microvascular networks are still of challenge.^[^ ^89^ ^]^ On the other hand, the whole volume of electrical tree channels is usually much smaller than the capacity of one single microcapsule (about 100 µm in diameter), suggesting that repeated healing is possible if new capsule structures are designed to enable multiple release of healing agents. Another issue associated with the extrinsic self‐healing methods is that incorporation of encapsulated healing agents is repulsive to the manufacturing process of thin films, extruded insulations and other finely structured dielectric components.

#### 3.1.2 Intrinsic Self‐Healing Methods

**Figure 4:**
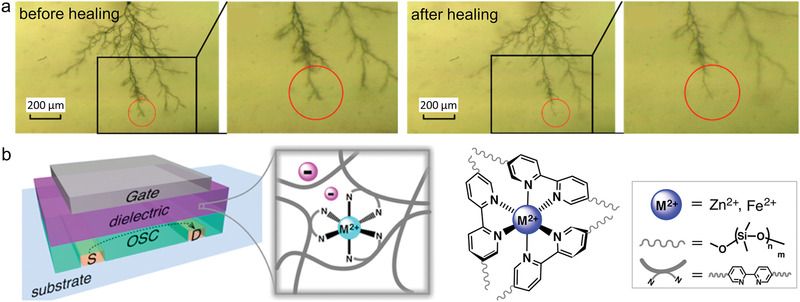
Previous tentative research and possible applications for intrinsic self‐healing dielectric polymer. a) Self‐healing of electrical tree branch tips in composite of hydrogen‐bonding self‐healing materials and epoxy matrix. Reproduced with permission.^[^ ^101^ ^]^ Copyright 2017, Elsevier. b) Schematics of the organic field‐effect transistor integrated with self‐healing metal‐ligand coordination cross‐linked polydimethylsiloxane. Reproduced with permission.^[^ ^107^ ^]^ Copyright 2016, American Chemical Society.

Intrinsic self‐healing materials can usually achieve multiple healing cycles of molecular level and microscale damage with/without external energy input such as heat, light and electromagnetic field.^[^ ^31^ ^]^ In general, the noncovalent self‐healing systems (hydrogel bond,^[^ ^90^, ^91^ ^]^ ionic interactions,^[^ ^92^, ^93^, ^94^ ^]^ etc.) and dynamic self‐healing materials ^[^ ^95^, ^96^ ^]^ can automatically repair damage without the input of external stimuli while the reversible (Diels–Alder bond,^[^ ^97^, ^98^ ^]^ metal‐ligand interactions,^[^ ^99^, ^100^ ^]^ etc.) and irreversible covalent bonding self‐healing networks usually need external energy to initiate the chemical reaction of healing. Bian et al. tried to develop a self‐healable composite by adding different hydrogen‐bonding self‐healing materials into the epoxy matrix.^[^ ^101^ ^]^ As shown in **Figure** **4**a, a minor decrease of tree branch tips was observed after a 2 h heating treatment. However, neither significant healing effect nor recovery of insulating properties were reported.

For the ionic and metal‐ligand interaction networks, which would be easily ionized and produce high leakage current under high electric fields, possible low‐voltage applications include flexible electronics,^[^ ^102^ ^]^ hydrogel ionotronics,^[^ ^103^ ^]^ soft robots,^[^ ^104^, ^105^ ^]^ organic field‐effect transistors,^[^ ^106^ ^]^ etc. Rao et al. incorporated metal‐ligand coordination into PDMS dielectrics as cross‐linking sites and endowed the polymer with fast self‐healing ability at the ambient condition.^[^ ^107^ ^]^ As is schematically illustrated in Figure 4b, the divalent metal cation cross‐linked PDMS is integrated into organic field‐effect transistors as gate dielectrics. The healing ability of metal‐ligand complexes upon electrical damage requires further assessment. In general, most of the intrinsic self‐healing thermoplastics can only repair damage above the glass transition temperature (*T*_g_) such that the polymer chains can flow into the crack regions and rebuild the networks. However, the mechanical properties of thermoplastic polymers usually vary abruptly near *T*_g_, which inhibits the self‐healing of materials when being in operation or possible applications requiring high mechanical performance.

### 3.2 Theoretical Considerations in Designing Self‐Healing Dielectric Polymers

Some basic healing performance of existing self‐healing materials including the healing scale range, maximum healing cycles, waiting time allowed between damaging and healing, chemical degradation tolerance and healing conditions (or working conditions and the accompanied effects during electrical damaging) are summarized in Table 1,^[^ ^31^, ^108^, ^109^ ^]^ where the corresponding features of electrical damage are listed for comparison.

The first challenge in healing electrical tree damage arises from its multi‐scale characteristic. In view of the existing self‐healing methods, most of them focus on the mechanical damage of specific length scale, such as micro‐cracks, scratches, and fractures. As the most extensively studied category of self‐healing strategies, intrinsic self‐healing via dynamic or reversible bonds is usually viable for healing of molecular and nanoscale fractures that remain in intimate contact ^[^ ^31^, ^36^ ^]^ while electrical trees are multi‐scale fractal damage with both nanoscale branch tips and microscale hollow tubules.^[^ ^75^ ^]^ The extrinsic methods, represented by microcapsule based self‐healing, cannot heal until the crack grows large enough (e.g., tens of micrometers or more) to reach and rupture the carriers of healing agent.^[^ ^31^ ^]^ It seems that the initial electrical trees, few‐micron‐long hollow tubes with nanoscale diameter, are beyond the length scale that most existing self‐healing methods can handle. Considering that the nanoscale electrical trees are mainly caused by Maxwell stress rather than partial discharge degradation, intrinsic self‐healing approaches based on dynamic chemistry may be promising for repairing the damage in the inception stage of electrical treeing. The extrinsic self‐healing approaches based on embedded healing agents possess potential in healing electrical tree damage during propagation stage. Nevertheless, ideal self‐healing dielectrics should be capable of repairing damage covering a broad range of length scales from nanometer to tens of micrometers and even millimeter. In thin film dielectric polymers, treeing processes before breakdown are usually too short to be observed thus some ionic rapid self‐healing mechanisms may be introduced for self‐healing of electrical breakdown.

Second, chemical degradation in long‐term electrical damaging and bulk breakdown brings up challenges especially for the intrinsic self‐healing methods which lack the chemical degradation tolerance. Electrical damaging is accompanied with chemical degradation (including thermal degradation,^[^ ^110^ ^]^ oxidative degradation,^[^ ^111^ ^]^ field ionization,^[^ ^112^ ^]^ photodegradation,^[^ ^113^ ^]^ hydrolysis ^[^ ^25^ ^]^ etc.) and physical destruction (including nonequilibrium stress by inhomogeneous electrical/thermal field, electro‐mechanical tearing ^[^ ^114^ ^]^ etc.) as a result of partial discharges. Therefore, different from mechanical damage that is usually caused by physical separation of tangled polymer molecules and chain rupture, in which cases the chemical component and properties of damaged regions remain nearly unchanged, electrical damage may cause chemical change in the damaged region and thereby destroy the dynamic functional components in most intrinsic self‐healing systems. In addition, the chemical degradation of polymers releases a large amount of low‐molecular‐weight byproducts such as amorphous carbon black, carbon oxide, hydrocarbon, and water,^[^ ^25^ ^]^ which introduce contamination defects in the damaged region. For the chemical byproducts, accumulation of the gaseous molecules forms microscale cavities and leads to local enhancement of mechanical and electrical stresses. This brings up more difficulties for self‐healing components to rebuild the damaged region.

Third, healing ability should be maintained after long waiting time (defined as the period of time from damaging to re‐contact of fracture surfaces) during the propagation stage of electrical tree damage. Most of the intrinsic self‐healing materials show reduced healing ability as the waiting time increases to hours.^[^ ^31^ ^]^ This is because the dangling reversible bonds exposed on the freshly damaged surface are likely to find co‐facial interaction partners over time and re‐associate within the broken parts rather than across the fractured surface.^[^ ^90^, ^94^, ^115^ ^]^ This phenomenon can be observed in most dynamic bonding networks if the reversible reactions are ready to proceed. Considering that electrical trees in high voltage electrical insulations are nearly invisible and hard to be detected timely, the healing process can only proceed during routine maintenance unless totally autonomous self‐healing of the electrical damage is developed. It is therefore challenging for dynamic bonding systems to repair this damage after long waiting time. For the extrinsic self‐healing methods, the damaged region can be healed as long as the agent can still flow into the tree channels and be solidified. However, the long‐term storage of healing agent is also a challenge in extrinsic self‐healing systems.^[^ ^31^ ^]^

Fourth, stringent performance requirements in high‐field and high‐temperature applications of dielectric polymers exclude most existing self‐healing systems. Although many self‐healing materials have been used as dielectrics in low‐electric‐field applications (e.g., organic flexible electronics) to repair mechanical damage ^[^ ^106^, ^116^ ^]^ or recover insulating properties from breakdown in thin films,^[^ ^117^, ^118^, ^119^ ^]^ which will be discussed in Section 4.4, few of them can be directly applied in high‐field electrical insulation because of their low dielectric strength. For most of the conventional self‐healing materials, electrical properties are unknown and only mechanical properties are reported. To make a qualitative comparison of dielectric strength between conventional self‐healing materials and high‐field dielectric polymers, the breakdown strengths of these materials are estimated based on the electromechanical breakdown model since they are usually of relatively low modulus^[^ ^120^ ^]^

(1) $$\begin{array}{c} E_{b}=0.606{\left[\frac{Y}{\varepsilon_{0}\varepsilon_{r}}\right]}^{1/2} \end{array}$$

where *E*_b_ is electromechanical breakdown strength, *Y* is the Young's modulus, *ε*_0_ is the permittivity of vacuum, and *ε*_r_ is the dielectric constant. The dielectric constant of many self‐healing materials is not available, and we set *ε*_r_ = 1 to give the maximum estimation of *E*_b_ which has negative correlation with *ε*_r_ according to Equation (1). Note that most of the dynamic self‐healing materials bearing a large amount of polar component possess high dielectric constant, the calculated values should be much higher than the real values. Because of the complex breakdown mechanism of dielectric polymers such as electronic breakdown and thermal breakdown, the theoretical *E*_b_ is usually 10 times higher than experimental values of breakdown strength ^[^ ^120^ ^]^ and the (*E*_b_/10) values of typical insulating self‐healing materials and conventional dielectric polymers are listed in Table 2 for comparison.^[^ ^4^, ^31^, ^108^, ^109^, ^121^, ^122^, ^123^, ^124^, ^125^, ^126^, ^127^ ^]^

For thin film dielectric polymers, stringent dielectric and thermal performance requirements in high‐field and high‐temperature applications leave limited material options. As shown in Table 2, except for some hydrogen bonding networks, the estimated breakdown strengths of most noncovalent intrinsic self‐healing materials are much lower than those of the conventional dielectric polymers. This is because the self‐healing ability of these materials rely on the high mobility of chain segments, and thus their mechanical strengths are much lower than common dielectric polymers. Besides, high mobility of chain segments also leads to low *T*_g_, which constrains the use of these materials in high temperature dielectric or insulation applications (Table 2). Although mechanically robust self‐healing materials based on hydrogen bonding networks have been reported,^[^ ^126^ ^]^ compression at fractured surfaces is necessary to complete the healing process. This is difficult to be implemented for healing of electrical tree damage because the tree branches are tubular hollow channels that are usually deep inside the material and are hardly spotted. Comparing with noncovalent bonding materials, reversible and irreversible covalent bonding self‐healing materials may possess higher dielectric strength and *T*_g_, but few is comparable to the commercial high temperature dielectric polymers. In general, existing intrinsic self‐healing methods have the potential to be used in thin film dielectric polymers under low electric fields and room temperature while application under high‐field and high‐temperature conditions is still challenging.

For bulk dielectric polymers, extrinsic healing agents are likely to increase the probability of electrical damage. Extrinsic self‐healing methods are generally applied in epoxy polymers which are widely used as bulk insulation in cable termination, bushing, dry‐type transformer, etc. In addition to the detrimental impact of metallic catalyst on dielectric properties of the composites, as has been reported in previous research,^[^ ^78^ ^]^ liquid healing agents also have notable adverse impact on the electrical resistivity and dielectric strength, which would increase the probability of electrical damage in long‐term operations. As shown in Table 2, extrinsic and ionic self‐healing systems generally exhibit low electrical resistivity which would lead to high dielectric loss in alternating current and energy storage applications. As a result, the key to the design of self‐healing dielectric polymers based on extrinsic methods is to achieve efficient repairing of electrical damage with minimized negative impact from the healing agents and catalysts.

## 4 Emerging Methods for the Self‐Healing of Electrical Damage

To overcome the challenges in developing self‐healing dielectric polymers, some new strategies have been designed in recent years to achieve restoration of insulating performance in common dielectric polymers.

### 4.1 Remolding of Multiscale Damage via Defect‐Targeted Superparamagnetic Heating

**Figure 5:**
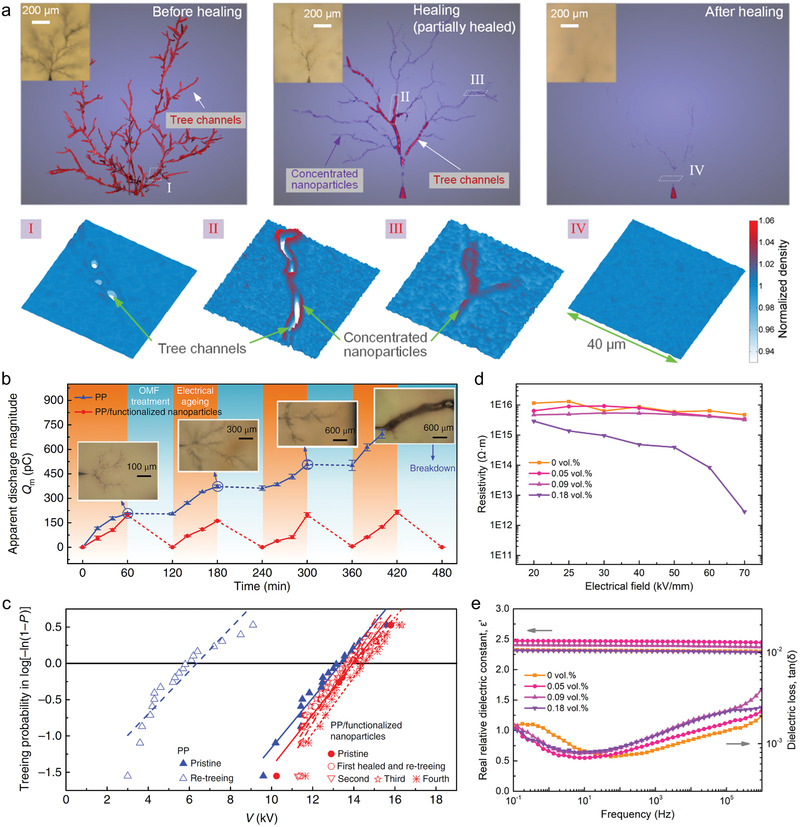
Self‐healing of electrical tree damages in thermoplastics using entropy‐driven defect‐targeted superparamagnetic heating approach. a) The 3D reconstruction of electrical trees in PP doped with 0.09 vol% functionalized superparamagnetic nanoparticles by computed micro‐X‐ray tomography before and after healing process. b) The maximum apparent discharge magnitude (*Q*_m_, indicating the partial discharge intensity or degradation degree) of pure PP and the self‐healing samples during the multiple ageing‐healing cycles. c) Weibull tree inception voltages of the samples suggesting repeated recovery of resistance to electrical aging in self‐healing samples. d,e) Basic electrical insulating performance, such as d) electrical resistivity and e) dielectric properties of the self‐healing dielectric polymers comparing with pure PP. Reproduced with permission.^[^ ^61^ ^]^ Copyright 2019, Nature Publishing Group.

Utilizing the magnetic heating effect and entropy‐driven defect‐targeted migration of superparamagnetic nanoparticles in polymers,^[^ ^128^, ^129^, ^130^ ^]^ Yang et al.^[^ ^61^ ^]^ developed a self‐healing approach capable of repeatedly repairing multiscale electrical tree damage (from nanoscale tree cracks to millimeter‐length tree channels) in thermoplastic polymers, e.g., PP, a common dielectric polymer used in power transmission cables, capacitors, etc. Surface‐functionalized superparamagnetic nanoparticles were incorporated into the polymer matrix and served as nanoscale heating units under oscillating magnetic field (OMF) through Néel and Brown relaxation effects.^[^ ^131^ ^]^ The surface functional layer around the nanoparticles was designed with specific thickness in order to 1) counteract van der Waals attraction between the particle cores, 2) induce sufficient conformational entropy penalty on the surrounding polymer chains,^[^ ^132^, ^133^, ^134^, ^135^ ^]^ and 3) maintain relatively high diffusion coefficient in the polymer melt. With these three requirements matched, the particles were observed to automatically migrate toward the surface of electrical tree channels, generating localized high temperature, and to re‐disperse after remolding the damaged regions (**Figure** **5**).^[^ ^61^ ^]^ Different from previous self‐healing research using superparamagnetic nanoparticles in which the edges of separated parts have to be put in good contact before healing,^[^ ^136^, ^137^, ^138^ ^]^ the emerging approach with defect‐targeted migration of the nanoparticles can repeatedly heal hollow tree channels and restore insulating properties. Interestingly, only less than 0.1 vol% of the superparamagnetic nanoparticles was found to be sufficient for the healing, which was attributed to the automatic and targeted transport and assembly of the nanoparticles toward the defect site. Therefore, the self‐healing dielectric polymers retain the electrical insulating performance (electrical resistivity, breakdown strength, tree inception voltage, etc.) of the pristine PP matrix (Figure 5d,e).

In addition to the healing of electrical damage, this defect‐targeted remolding approach has also been used in the healing of different damage modes such as cracking and puncturing in various thermoplastics (PMMA and perfluorosulfonic acid) and in the restoration of different functionalities including mechanical strength and electrical conductivity.^[^ ^139^ ^]^ The effectiveness of this approach is also independent of the waiting time after the formation of defect,^[^ ^139^ ^]^ which represents another advantage over the conventional intrinsic or extrinsic self‐healing strategies in the repairing of electrical damage. The need for OMF as the external stimulus would increase the complexity of this healing approach, but in some applications this may not be an issue because the near‐wire magnetic field generated by standard electrical equipment such as power electronic modules and wireless charging systems is comparable to the OMF employed,^[^ ^61^ ^]^ which could facilitate the self‐healing when the dielectrics are in operation. For the application in power transmission systems, a periodically scheduled injection of oscillating current from convertor stations can be performed to create the OMF required for the self‐healing and maintenance of the insulating layer. As many kinds of inorganic insulating nanoparticles have been used to improve dielectric performance of polymers in various electrical insulation applications,^[^ ^59^, ^140^, ^141^, ^142^, ^143^ ^]^ it is promising to develop hybrid magnetic healing nanoparticles with superparamagnetic core and insulating shell to endow dielectric polymers with both self‐healing functionality and enhanced insulating performance.

### 4.2 Autonomous Self‐Healing of Electrical Tree using In Situ Electroluminescence

**Figure 6:**
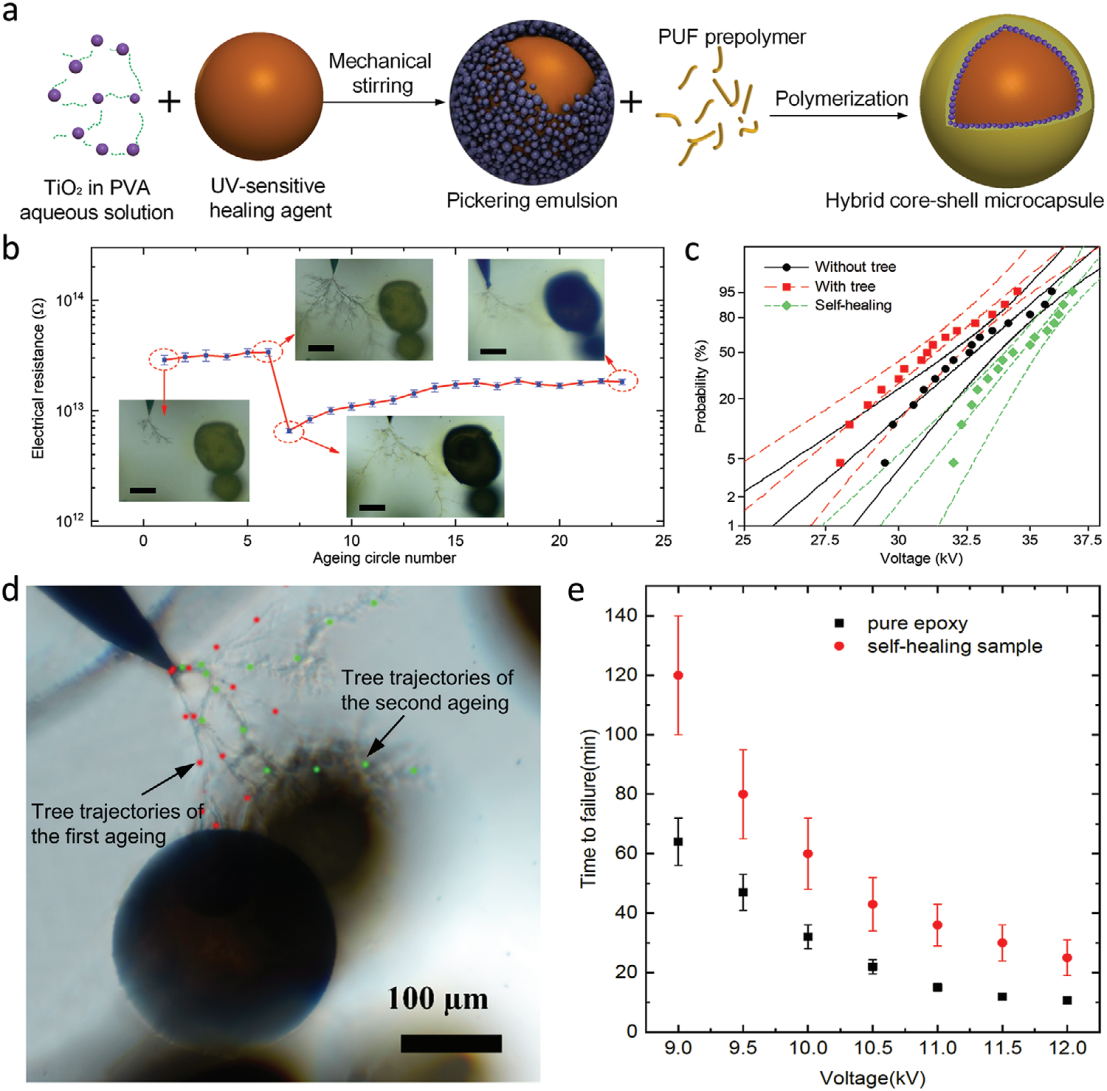
Self‐healing of electrical tree damages in thermosets using microcapsules and in situ electroluminescence. a) Schematics of preparation of the hybrid microcapsules with UV‐shielding PUF/TiO_2_ shell. b) Direct‐current electrical resistance measured in a typical degradation and healing curve of the self‐healing polymer. Scale bar, 100 mm. c) Weibull breakdown voltage with 90% confidence limits of the self‐healing samples with/without tree defect and after healing process, suggesting improved local insulating properties of the healed region. d) Electrical tree trajectories of the re‐treeing process after healing. e) Electrical aging lifetime of pristine epoxy and self‐healing samples. Reproduced with permission.^[^ ^62^ ^]^ Copyright 2019, Cell Press.

Self‐healing approaches based on embedded healing agents can heal microscale damage in the propagation stage of electrical tree. To eliminate the adverse effect of catalysts on the insulating performance, Gao et al.^[^ ^62^ ^]^ designed a catalyst‐free microcapsule‐based self‐healing strategy utilizing in situ electroluminescence (UV‐light emission) generated by the electrical treeing process to trigger the curing of healing agents (**Figure** **6**). To minimize the detrimental impact of liquid healing agents on the electrical insulating properties of the polymer matrix, the composites were designed with dielectric inhomogeneity, which guides the propagation of electrical tree toward the nearest microcapsule and enables relatively low addition of the healing agent. Furthermore, SiO_2_ nanoparticles were mixed with the healing agents to reduce the negative impact of the microcapsule on the electrical insulating properties and the self‐healing composites maintained about 90% dielectric breakdown strength of the pristine epoxy polymer.

To realize long term storage of healing agent for repeated self‐healing, an UV‐shielding shell of the microcapsule was fabricated by incorporating TiO_2_ nanoparticles into the poly(urea‐formaldehyde). This hybrid shell would protect the encapsulated healing agent from the electroluminescence generated during treeing until the microcapsule is ruptured and the healing agent fills the tree channels. The healed regions exhibit high electrical insulating performance after curing, which enables enhanced local breakdown strength and prevents secondary damage at the same region. As shown in Figure 6d, the secondary treeing, initiated by increased inception voltage, show different trajectories of propagation. According to the electrical aging test and lifetime evaluation based on the fluctuation model, the self‐healing composites show improved ability to withstand electrical degradation and extended service lifetime with respect to the pristine polymer. This approach is feasible for the healing of thermoset dielectric polymers.

### 4.3 Self‐Healing Dielectric Polymers via Anionic Polymerization

**Figure 7:**
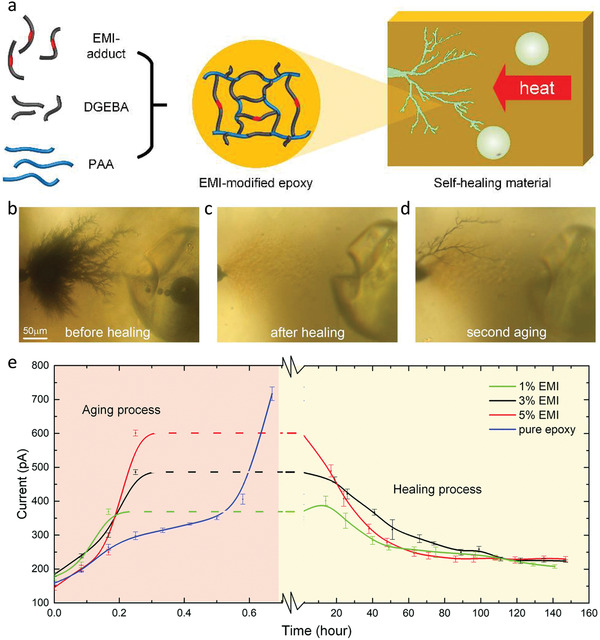
Self‐healing of electrical tree damages in thermosets using anionic polymerization‐based microcapsule approach. a) Schematic synthesis and mechanism of self‐healing material. Microcapsules were added into the EMI‐modified epoxy to give the material self‐healing ability. b–d) Electrical tree trajectories b) before and c) after healing followed by d) a secondary aging process, in which the tree trajectories bypass the healed regions. e) The leakage currents of typical self‐healing samples with different concentrations of EMI during the aging and healing process. Reproduced with permission.^[^ ^63^ ^]^ Copyright 2020, Royal Society of Chemistry.

The self‐healing approach using in situ electroluminescence requires that the dielectric polymers to be healed are transparent to the UV light, but thermoset insulations in practical applications can be opaque to the UV light because of the embedded additives. To address this issue, Xie et al.^[^ ^63^ ^]^ designed an anionic polymerization‐based microcapsule approach. As illustrated in **Figure** **7**, the epoxy matrix is modified with 2‐ethyl‐4‐methylimidazole (EMI). Diglycidyl ether of bisphenol A (DGEBA) is selected as the healing agent monomer having low reactivity with EMI‐adduct at room temperature, so that the anionic polymerization reaction between EMI‐modified epoxy and the healing agent is selectively activated at a moderate temperature range matching with the working condition of many polymer insulations in electrical power systems.^[^ ^63^ ^]^ A low content of EMI (1–3 wt%) is introduced into the epoxy molecular chain to form the matrix of self‐healing polymer while maintaining the excellent insulating and mechanical properties of the materials. The electrical tree channels can be healed without any additional stimuli and complete recovery of dielectric properties are demonstrated through leakage current and partial discharge tests. Other existing studies using anionic polymerization to fabricate self‐healing materials focus on mechanical damages, such as epoxy‐based binary self‐healing systems^[^ ^144^, ^145^, ^146^, ^147^, ^148^, ^149^ ^]^ and reversible crosslinking of block copolymers.^[^ ^150^ ^]^ Generally, anionic polymerization is conducted in a harsh condition to avoid side reactions which deactivate the living anionic ions, which in turn favors the self‐healing of electrical insulations operating at elevated temperatures. By employing different polymerization initiators and healing agent monomers, the anionic polymerization reaction can be selectively activated at 80–180 °C, which provides opportunities to develop various self‐healing dielectric polymers matching different working conditions in the field of high‐voltage polymer insulations. For example, in the semiconductor encapsulation materials of power electronics module, the polymer insulation operates at maximum temperature of about 165–200 °C for polyurethane‐ and epoxy‐based medium‐temperature applications.^[^ ^151^ ^]^ The self‐healing of such insulations may be accomplished with the anionic polymerization reactions using hardeners of the epoxy healing agent such as 2,4‐diamino‐6‐[2’‐methylimidazolyl‐(1)’]‐ethyl‐triazine (2MZ‐AZINE) for ring‐opening polymerization of epoxy at 120–150 °C,^[^ ^146^ ^]^ 2‐ethyl‐4‐methylimidazole (24‐EMI) that is usually used at 140 °C,^[^ ^145^ ^]^ and CuBr_2_(2‐MeIm)_4_ (the complex of CuBr_2_ and 2‐methylimidazole) which can be activated at 130–180 °C.^[^ ^149^ ^]^

One of the major problems faced by extrinsic self‐healing methods is the instability of the healing agent in long term storage. Conventional unsaturated healing agents or monomers are prone to self‐polymerize in long‐term storage, especially at high temperature conditions. In the anionic polymerization‐based approach, the bisphenol A epoxy resin possessing high thermal stability is adopted as the healing agent. Accelerated thermal aging experiments demonstrate the long‐term effectiveness of the healing agent and EMI‐modified epoxy, which allows for long‐running and high‐temperature insulation applications. However, for all the extrinsic self‐healing methods, the adverse impact of liquid healing agent on dielectric properties still brings up challenges in long‐term electrical insulation. Moreover, gaseous byproducts generated by electrical degradation during the treeing process will accumulate and form voids inside the microcapsules or vascular fibers, and act as insulating defects. In general, although these new designs have overcome some bottleneck issues of self‐healing dielectric polymers, various problems remain to be resolved.

### 4.4 Self‐Healing of Electrical Breakdown in Polymer Thin Films

**Figure 8:**
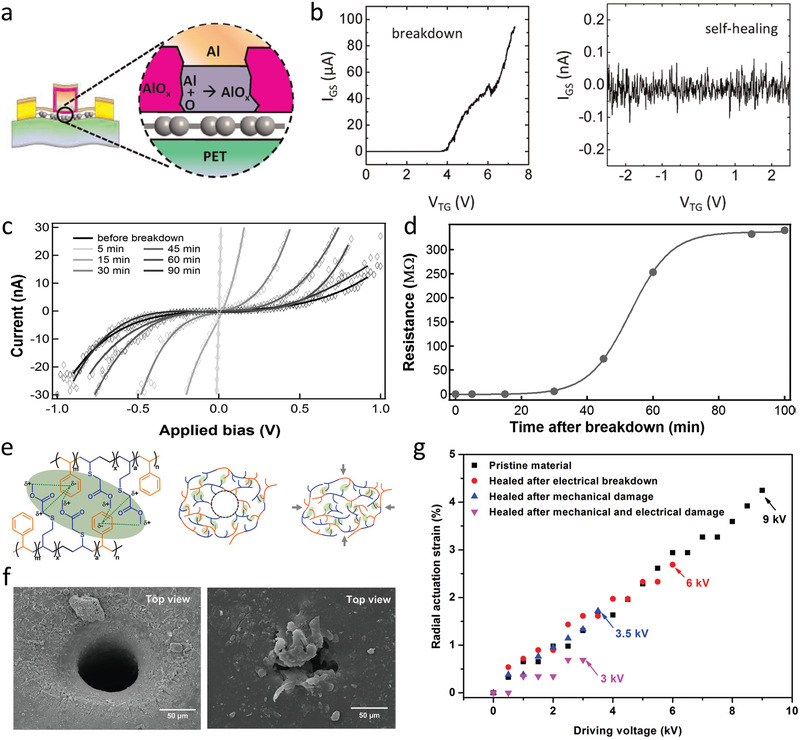
Self‐healing of electrical breakdown in dielectric films. a) Schematic of the self‐aligned graphene field‐effect transistor using monolayer aluminum oxide as a gate dielectric, showing the regrowth of native AlO*_x_* at the breakdown site. b) The *I*_GS_–*V*_TG_ curves after breakdown and the following self‐healing process, indicating recovery of electrical resistance of the AlO*_x_* layer. a,b) Reproduced with permission.^[^ ^153^ ^]^ Copyright 2012, American Chemical Society. c) *I*–*V* curves of a lipid monolayer dielectric at different time after electrical breakdown and d) the extracted evolution of electrical resistance at 1 MV cm^−1^. c,d) Reproduced with permission.^[^ ^154^ ^]^ Copyright 2011, American Chemical Society. e) Schematics of an elastomer network as the self‐healing dielectric actuator and the healing mechanism with the *δ* ^+^ proton adjacent to the ester interacting with the *δ* ^−^ aromatic center of styrene. f) Scanning electron microscopy images of the breakdown site after cleaning of the carbonized area (left) and followed by application of pressure with fingers as the healing treatment. g) Radial actuation strain of the pristine elastomer and healed elastomer which experienced secondary breakdown at 67% (6 kV) of initial breakdown strength. e–g) Reproduced with permission.^[^ ^119^ ^]^ Copyright 2019, Wiley‐VCH.

“Self‐healing” film capacitor dielectrics possess the ability to tolerate electrical breakdown by evaporating the metal electrodes of typically a few mm^2^ around the breakdown site rather than forming permanent short‐circuit.^[^ ^37^ ^]^ In this case, the dielectric polymers themselves are not healable and the electrical breakdown sites are still permanent damage thus the “self‐healing” behavior is more often referred to as self‐clearing.^[^ ^152^ ^]^ As the “real” self‐healing dielectrics, various ultrathin monolayers (few nanometers in thickness) ^[^ ^153^, ^154^, ^155^ ^]^ (**Figure** **8**a–d) and thin film polymers (less than tens of micrometers in thickness) ^[^ ^117^, ^118^ ^]^ can automatically heal breakdown sites with or without reversible bonds. Taking the ionic cross‐linked self‐healing network as an example, the breakdown hole of the thin film is so small (smaller than 10 µm) that the ionic parts are able to flow into the hole and heal the materials at elevated temperature.^[^ ^118^ ^]^ A dielectric elastomer was reported to be capable of healing electrical breakdown holes in 510 µm thick films by the intermolecular electrostatic interaction between the methyl thioglycolate‐modified butadiene block and the styrene block.^[^ ^119^ ^]^ As shown in Figure 8e–g, because of the large hole size of breakdown site (about 50 µm in diameter), self‐healing of the damage requires application of pressure with fingers and only less than 67% of initial breakdown strength can be restored.^[^ ^119^ ^]^

Among all the studies on the self‐healing of catastrophic breakdown utilizing dynamic bonding chemistries, the maximum electrical breakdown strength of the self‐healing dielectric polymers is around 50 kV mm^−1^, much lower than the commonly used dielectric polymers, e.g., biaxially oriented PP (>700 kV mm^−1^), LDPE (>350 kV mm^−1^), and poly(ether imide) (PEI, >400 kV mm^−1^).^[^ ^123^ ^]^ Electrical breakdown in bulk dielectric polymers of high breakdown strength usually results in large‐area ablation and carbonization through the breakdown pathway, and produces a large amount of semi‐conductive degradation byproducts on the surface of the breakdown hole, bringing difficulties to the healing of the damaged region.

## 5 Potentially Viable Mechanisms for Developing Novel Self‐Healing Dielectric Polymers

Despite the aforementioned challenges in the design of self‐healing dielectric polymers, the special working conditions (i.e., multiple stimuli and energy sources^[^ ^156^ ^]^) of dielectric polymers and various concomitant phenomena during electrical degradation provide opportunities for future development. This section proposes some mechanisms and methods, although none of them has been used to heal electrical damage in polymers that are potentially viable for developing new self‐healing dielectric polymers.

### 5.1 Absorption and Clearing of Electrical Degradation Byproducts

**Figure 9:**
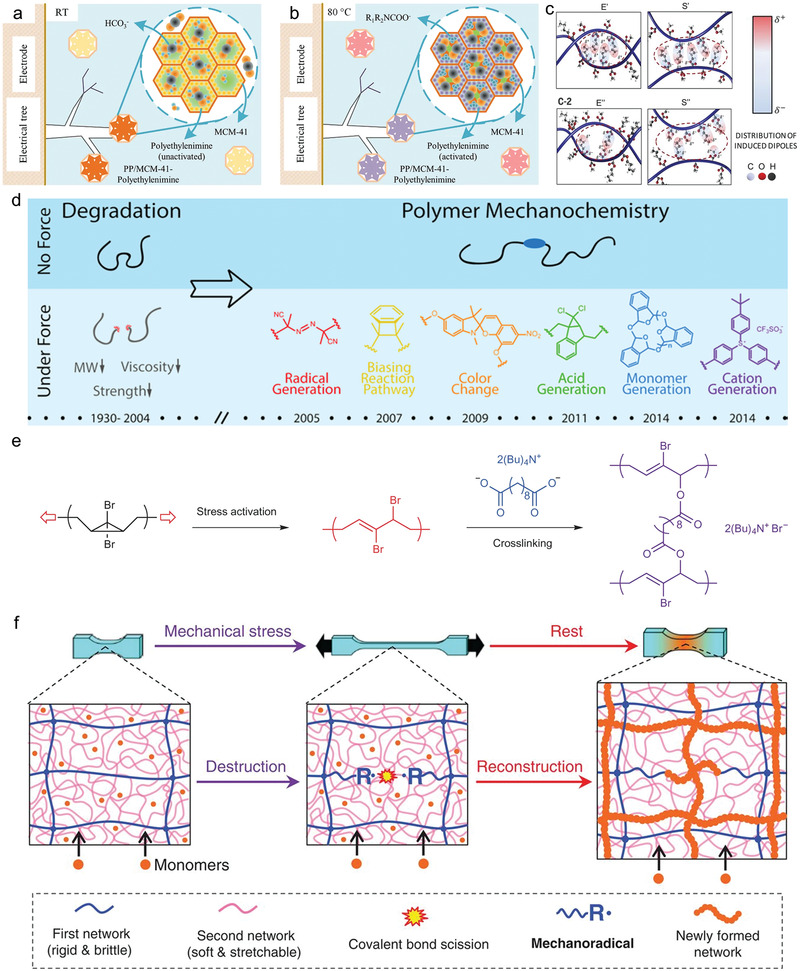
Potentially viable mechanisms for developing novel self‐healing dielectric polymers. a,b) Schematics of the degradation inhibitors capable of absorbing gaseous electrical degradation products in electrical tree channels by a) hydrolysis (produces bicarbonate) and b) zwitter‐ion reaction (produces carbamate) at room temperature (RT) and 80 °C respectively. Reproduced with permission.^[^ ^58^ ^]^ Copyright 2016, Nature Publishing Group. c) Self‐healing of “key‐and‐lock” copolymers by interchain van der Waals forces. Pictorial representation of the distribution of induced dipole moments in self‐healable entangled (E’) and side‐by‐side (S’) chains. Pictorial representation of distribution of induced dipole moments for none self‐healable entangled (E’’) and side‐by side (S’’) chains. Reproduced with permission.^[^ ^162^ ^]^ Copyright 2018, American Association for the Advancement of Science. d–f) Mechanochemical interactions in self‐strengthen and self‐growing materials. d) Pioneering works in polymer mechanochemistry reported by Moore group. Reproduced with permission.^[^ ^183^ ^]^ Copyright 2015, American Chemical Society. e) Self‐strengthen polymer based on a mechanochemical ring‐opening reaction. Reproduced with permission.^[^ ^188^ ^]^ Copyright 2013, Nature Publishing Group. f) Schematics of a biological metabolism‐inspired self‐growing double‐network hydrogel based on a radical‐generating mechanochemical reaction. Reproduced with permission.^[^ ^189^ ^]^ Copyright 2019, American Association for the Advancement of Science.

The low‐molecular‐weight degradation byproducts generated in the electrically damaged region would accelerate the aging process and impede healing of the materials. Yang et al. developed the degradation inhibitors capable of absorbing gaseous electrical degradation byproducts (including CO_2_ and H_2_O) in electrical tree channels.^[^ ^58^ ^]^ As illustrated in **Figure** **9**a,b, the polyethylenimine loaded nanoscale mesoporous silica MCM‐41 particles are doped into PP matrix. The abundant amine groups in the polyethylenimine capture the electrical degradation byproducts while the MCM‐41 brackets afford large adsorption surface, decreasing the activation temperature of polyethylenimine and improving the absorption capacity.^[^ ^58^ ^]^ Hydrolysis of CO_2_ can be catalyzed by amino groups and produce bicarbonate at room temperature. For direct stable sequestration of CO_2_, zwitter‐ion reaction can be activated at high temperature that is comparable to the working condition of dielectrics in high‐temperature applications.^[^ ^58^ ^]^ In addition, the organic amine functionalized mesoporous silica nanoparticles MCM‐41‐polyethylenimine can greatly improve the electrical insulating properties including electrical breakdown strength,^[^ ^58^ ^]^ resistivity and threshold field of space charge injection,^[^ ^157^ ^]^ which is crucial for extruded DC power cable and many other high‐field insulating applications.^[^ ^158^ ^]^

The degradation inhibitors can be introduced into the self‐healing systems to absorb gaseous electrical degradation byproducts so that the self‐healing components can work. For the solid byproducts (such as amorphous carbon black) produced by ablation and carbonization around the wall of electrical tree channels and breakdown holes, it is difficult to be cleared through chemical reaction. A possible approach is to employ physical penetration paths to deliver active healing agents and cover the surface of damaged regions, which will be discussed in the next section.

### 5.2 Interpenetrating Polymer Networks

Interpenetrating polymer networks (IPNs) are generally thermoset‐thermoplastic composites with certain percentages of the linear polymer phase interpenetrated into the cross‐linked polymer phase.^[^ ^159^ ^]^ Self‐healing using the thermoset‐thermoplastic composites include highly miscible systems ^[^ ^160^ ^]^ and phase‐separated systems ^[^ ^161^ ^]^ with the reported healing efficiencies up to 77% and 100%, respectively. In the IPN systems, it is reported that diffusion of linear polymer into the crack interface could heal the crack upon heating.^[^ ^159^ ^]^ Although the diffusion of linear polymer is limited to micrometer scale and is unable to heal large open cracks, this approach may be utilized to heal the initial trees and to clear the carbonized inert surface of the tree channel walls created by severe partial discharges. As demonstrated by Soxhlet extraction, the linear polymer phase is mobile within the IPN and enables chain interdiffusion and entanglement that brings about temperature‐ and time‐dependent enrichment of the surface.^[^ ^159^ ^]^ This surface enrichment (or cleaning) process may assist the intrinsic self‐healing approaches against electrical degradation and improve the healing efficiency.

Interdiffusion and entanglement of thermoplastic chains in the healing process of IPNs are driven by Brownian motion and intermolecular van der Waals interaction, which are highly related to the conformation and topology of polymer chains. Urban et al. reported similar self‐healing behaviors in poly(methyl methacrylate)/n‐butyl acrylate [p(MMA/nBA)] and their derivatives in response to mechanical damage.^[^ ^162^ ^]^ As illustrated in Figure 9c, the healing behavior is attributed to key‐and‐lock interchain junctions formed by favorable interchain van der Waals forces within a narrow compositional range. Copolymers with alternating/random topologies preferentially form high density of “key‐and‐lock” interactions of interdigitating alkyl pendant groups between chains, and creates a viscoelastic response that energetically favors self‐healing upon chain separation.^[^ ^162^ ^]^ This new self‐healing mechanism is promoted by nanoscale design of molecular movement and phase separation,^[^ ^163^ ^]^ eliminating chemical and physical alterations ^[^ ^162^ ^]^ and the dependence of self‐healing functionalities on specific chemical components, which possesses potential for healing electrical damage in dielectric polymers.

### 5.3 Automatic Crack Closure

Lendlein et al. synthesized an AB‐polymer network with shape memory properties.^[^ ^164^ ^]^ Generally, the shape memory polymers (SMPs) show at least two separated phases with the higher thermal transition phase standing for the permanent shape below *T*_perm_ and the lower thermal transition phase working for the fixation of temporary shape below the switching temperature *T*_trans_.^[^ ^165^ ^]^ By heating up the material above *T*_trans_ but below *T*_perm_, the temporary shape will be destroyed, and the permanent shape could be recovered. SMPs would introduce shape memory effect into many easy shaping, light‐weight, and flexible transition temperature areas,^[^ ^164^ ^]^ and shape memory assisted self‐healing (SMASH) has been attracting more attention.^[^ ^166^, ^167^, ^168^, ^169^ ^]^ Rodriguez et al. reported a SMASH system that is able to close and bond cracks simultaneously with thermal trigger.^[^ ^170^ ^]^ This strategy is realized by a blend system consisting of cross‐linked poly(*ε*‐caprolactone) (n‐PCL) network and linear poly(*ε*‐caprolactone) (1‐PCL), which combines the shape memory property of the network and the self‐healing function of the linear component together. With the assistance of the shape memory epoxy matrix, they also developed the SMASH coating containing electro‐spun PCL fibers capable of flowing into and rebuilding the crack regions upon heating.^[^ ^171^ ^]^ Similar works were reported by Zhao et al.^[^ ^172^ ^]^ Besides the thermally triggered SMPs, some other excitations such as optical,^[^ ^173^, ^174^, ^175^ ^]^ electrical,^[^ ^176^, ^177^ ^]^ magnetic,^[^ ^178^ ^]^ and chemical ^[^ ^179^, ^180^, ^181^ ^]^ ones were used to develop the SMASH materials. Multi‐responsive SMPs provide a variety of damage‐induced or stimuli‐responsive functionalities that could potentially assist the self‐healing of electrical tree and breakdown damage.

Other driving mechanisms that can be utilized to achieve automatic crack closure include conformational entropy and interfacial energy. Hornat and Urban developed entropy and interfacial energy driven self‐healable polymers capable of repairing without intervention.^[^ ^182^ ^]^ Two self‐healing mechanisms are identified depending on the molecular weight of the polymer networks. Viscoelastic shape memory driven by conformational entropic energy stored during mechanical damage enables sufficient molecular mobility for self‐healing while maintaining high strength and stiffness through precise material design. For low molecular weight polymers, surface energy/tension can drive the reduction of newly generated surface areas created upon damage by shallowing and widening wounds until healed. These two autonomous self‐healing mechanisms result from the viscoelastic behavior of polymers irrelevant to the chemical components,^[^ ^182^ ^]^ and they could potentially serve as general approaches possessing robust healing ability upon chemical degradation in electrical damage.

### 5.4 Mechanochemical Self‐Strengthening and Self‐Growing Networks

Electrical damaging is always accompanied by localized enhancement of Maxwell stress that causes mechanical rupture of polymer chains. Mechanochemical reactions are expected to respond to the electrostatic stress, dissipate the mechanical strain energy and spontaneously strengthen the forefront tree branch regions so that the damaging process would be slowed down and yield to the healing process. The Moore group has reported many pioneering works in polymer mechanochemistry, as shown in Figure 9d.^[^ ^183^ ^]^ Other studies referred these constructive bond‐forming reactions as activated remodeling via mechanochemistry (ARM) and used it to produce new chemistry,^[^ ^184^ ^]^ force accelerated and selective transformations,^[^ ^185^ ^]^ self‐strengthening or self‐healing polymers,^[^ ^186^, ^187^ ^]^ etc. Ramirez et al. demonstrated that mechanochemical reactions can be used to strengthen a polymer subjected to the same shear forces responsible for destructive processes (Figure 9e).^[^ ^188^ ^]^ The dibromocyclopropane mechanophores can generate allylic bromides by mechanical activation, which are cross‐linked in situ by nucleophilic substitution reactions with carboxylates bringing orders‐of‐magnitude increases in bulk modulus.^[^ ^188^ ^]^

For the tree channels and breakdown holes, mechanochemical self‐growing reactions provide the opportunities to make up for the material loss after electrical degradation by self‐growing. Recently, Matsuda et al. proposed a biological metabolism‐inspired strategy to develop self‐growing materials in response to mechanical stress through a mechanochemical transduction.^[^ ^189^ ^]^ As shown in Figure 9f, upon mechanical stress, the double‐network hydrogels generate mechanoradicals at the broken ends of the brittle network strands and trigger the polymerization of monomers supplied from the external environment.^[^ ^189^ ^]^ This method endows the polymer matrix with the ultimate self‐healing ability in certain solution environments, which could potentially be utilized to regenerate electrical degraded regions in aquatic and liquid‐containing electrical devices such as electroactuators,^[^ ^190^ ^]^ hydrogel ionotronics,^[^ ^103^ ^]^ and poly(ionic liquid)s supercapacitors.^[^ ^191^ ^]^

### 5.5 Multi‐Stress Coupled Stimuli‐Responsive Materials

**Figure 10:**
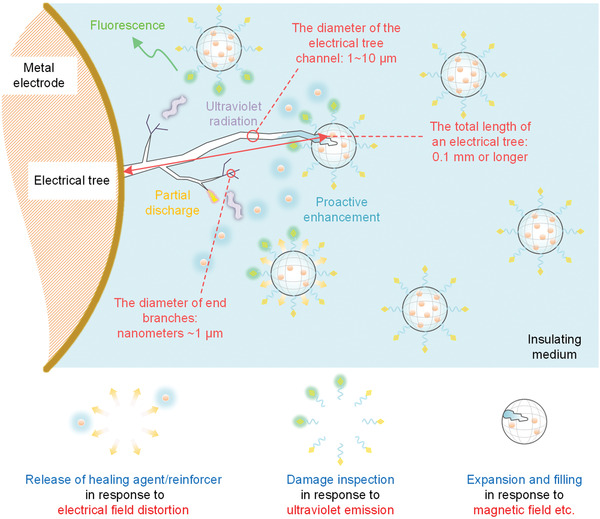
Conceptual design of self‐healing insulation system including automatic closing of tree channel, release and storage of healing agent/reinforcer storage and micro‐damage detection using multi‐stress‐coupled stimuli‐responsive materials.

After Tanaka discovered the “volume phase transition” phenomena (gels change volume reversibly in response to environmental changes),^[^ ^192^ ^]^ gel materials have attracted extensive research interest. Some polymer gels can cause bending motion and swelling‐deswelling changes in response to external stimuli including temperature change,^[^ ^193^, ^194^ ^]^ light irradiation,^[^ ^195^ ^]^ electric field,^[^ ^196^, ^197^ ^]^ magnetic field,^[^ ^198^ ^]^ etc. Many smart materials based on the stimuli‐responsive polymer gels have been developed for applications such as artificial muscles, drug delivery systems and biosensors.^[^ ^198^, ^199^, ^200^, ^201^, ^202^ ^]^ These smart materials can be divided into three categories: mechanical motion, mass transport, conversion and transmission of information,^[^ ^200^ ^]^ which could potentially assist the self‐healing of electrical damage by automatic closure of tree channels, storage/release of healing agents and detection of micro‐damage respectively, as illustrated in **Figure** **10**.

In this conceptual design, stimuli‐responsive polymers may be utilized as the shell material to compose smart microcapsule systems. The smart microcapsules may respond to the electric field distortion, magnetic field and ultra‐violet radiation of partial discharge and then proactively release the embedded healing agent/reinforcer to repair/strengthen the damaged region. For example, using electrokinetic molecular assembly reaction of surfactant molecules ^[^ ^196^ ^]^ on the inner wall of the capsule shell, the microcapsule shells could act as electrically driven actuators and release healing agent/reinforcer to the damaged region where the electric field is enhanced. Like the drug delivery systems, these smart microcapsules are expected to be more sensitive to the initial damage comparing with the conventional microcapsules and the release of healing agent is controlled by the degree of electrical degradation. In addition to self‐healing, the multi‐stress coupled stimuli‐responsive strategies may be used to trigger the intelligent insulating composite systems to implement damage reporting, adaptive strengthen and other functionalities, which provide opportunities to design smart dielectric polymers for electronics and energy applications.

## 6 Summary and Outlooks

Self‐healing is becoming one of the integral functionalities of materials in many technical fields including construction industry (self‐healing concrete),^[^ ^203^, ^204^, ^205^ ^]^ military (bullet‐holes self‐healing materials),^[^ ^92^ ^]^ auto industry (scratch‐resistant coating),^[^ ^206^ ^]^ and so forth. However, few self‐healing strategies have been introduced to the electronics and electric power industry. Witnessing the fast growth of demand in electric energy and electronic devices, we expect that self‐healing electrical insulating systems will play significant roles and possess wide application prospects.

The multi‐scale characteristic of electrical damage, complex electrical aging process, and harsh operating conditions bring great challenges in designing self‐healing dielectric polymers. Conventional self‐healing strategies using embedded healing agent will introduce inhomogeneity and insulation defects into the matrix, which may cause local stress enhancement under electric field and increase the probability of electrical damaging. Although improved microcapsule self‐healing epoxy composites using electroluminescence and anionic polymerization have been developed,^[^ ^62^, ^63^ ^]^ conventional fabrication methods of dielectric polymers need to be modified to accommodate the incorporation process of microcapsules, and the long‐term storage of healing agent is also challenging. Regarding intrinsic self‐healing systems, chemical degradation of the dielectrics during electrical aging may destroy the healing functionalities and impede necessary intimate contact of fracture surfaces. Thus clearing of degradation byproducts, enrichment/cleaning of carbonized damage surface and automatic closure of electrical tree channels or breakdown holes should be considered as auxiliary functionalities in self‐healing dielectric systems. Possible solutions include employing degradation product absorbent, interpenetrating self‐healing networks, conformational entropy and surface energy mechanisms in self‐healing dielectric polymers. As the thermoplastic extruded insulation cables are getting more and more popular, the entropy‐driven defect‐targeted superparamagnetic heating approach ^[^ ^61^ ^]^ shows promise in self‐healing power transmission systems. Stimuli‐responsive polymer gels provide various opportunities in designing smart dielectric systems capable of automatic closure of tree channels, storage/release of healing agents and micro‐damage detection under the multi‐stress‐coupled electrical aging conditions. Mechanochemical self‐strengthening and self‐growing mechanisms provide opportunities for assisting the self‐healing process in competition with the damaging process and achieving ultimate self‐healing ability making up for the material loss in catastrophically damaged regions.

Self‐healing of electrical damage in dielectric polymers is an emerging frontier area that is of major difference to the conventional research regarding dielectric and insulation materials, and that significantly broadens the self‐healing concept from repairing mechanical fracture to electrical damage in polymers. The ability to completely restore the dielectric strength and electrical properties of dielectric polymers from electrical tree degradation and catastrophic breakdown offers unprecedented opportunities for the development of smart polymers with greatly increased lifetime, safety and sustainability for electronics and energy applications, which may lead to revolutionary progress in the electronics and electric power industry. The success in this challenging yet promising research field will need interdisciplinary collaboration that brings conceptual advance in dealing with the stringent set of requirements in developing self‐healing dielectric polymers.

## Conflict of Interest

The authors declare no conflict of interest.
